# Gamma analysis with a gamma criterion of 2%/1 mm for stereotactic ablative radiotherapy delivered with volumetric modulated arc therapy technique: a single institution experience

**DOI:** 10.18632/oncotarget.18530

**Published:** 2017-06-16

**Authors:** Jung-in Kim, Minsoo Chun, Hong-Gyun Wu, Eui Kyu Chie, Hak Jae Kim, Jin Ho Kim, Jong Min Park

**Affiliations:** ^1^ Department of Radiation Oncology, Seoul National University Hospital, Seoul, Republic of Korea; ^2^ Institute of Radiation Medicine, Seoul National University Medical Research Center, Seoul, Republic of Korea; ^3^ Biomedical Research Institute, Seoul National University College of Medicine, Seoul, Republic of Korea; ^4^ Department of Radiation Oncology, Seoul National University College of Medicine, Seoul, Republic of Korea; ^5^ Center for Convergence Research on Robotics, Advanced Institutes of Convergence Technology, Suwon, Republic of Korea

**Keywords:** 2D gamma evaluation, pre-treatment patient-specific quality assurance, volumetric modulated arc therapy, stereotactic ablative radiotherapy, gamma criterion

## Abstract

To report a single-institution experience of gamma evaluations with 2%/1 mm for stereotactic ablative radiotherapy (SABR) delivered with volumetric modulated arc therapy (VMAT) technique, from January 2014 to January 2016. A total of 168 SABR VMAT plans were analyzed with a gamma criterion of 2%/1 mm, a threshold value of 10%, and a tolerance level of 90%. Of the 168 cases, four cases failed with 2%/1 mm. The average passing rate was 97.0% ± 2.5%. Three of the four failed cases showed passing rates higher than 90%, which was achieved by shifting the measuring device by 1 mm in the left-to-right or anterior-to-posterior directions. One failed case showed a passing rate higher than 90%, which was achieved by changing the threshold value from 10% to 5%, leading to an increase in the number of tested points from 26 to 51. Concerns regarding the high susceptibility of the gamma criterion of 2%/1 mm to setup errors of the measuring device are unnecessary based on our two-year experience, since only four cases failed with the 2%/1 mm from a total of 168 clinical cases. Therefore, the gamma criterion of 2%/1 mm could be successfully applied in the clinic with its high sensitivity to detect errors in VMAT plans.

## INTRODUCTION

Intensity modulated radiation therapy (IMRT) as well as volumetric modulated arc therapy (VMAT) can deliver highly conformal prescription doses to target volumes while minimizing doses to organs at risk (OARs) in proximity to the target volumes, which enables high local control as well as reduction of complications related to radiotherapy [1–5]. This could be achieved by using the inverse planning algorithm and modulations of photon beam intensities [6, 7]. In the case of IMRT, the photon beam intensities are modulated with superpositions of photon beams with various beam apertures defined by the multi-leaf collimators (MLCs), *i.e.*, static IMRT, or movements of each MLC with various speeds during beam-on time, *i.e.*, dynamic IMRT [8, 9]. In rare instances, IMRT could be delivered with compensators manufactured for each field at the institutions in which an MLC system was not available [10, 11]. On the contrary, for VMAT, the photon beam intensities are modulated with simultaneous modulations of three parameters: the gantry rotation speeds, dose-rates, and MLC positions [7, 12]. For both the IMRT and VMAT, high modulation of photon beam intensities may generate a clinically better quality treatment plan; however, it could cause discordance in dose distributions between the calculated dose in the treatment planning system (TPS) and the actual dose delivered to a patient during treatment [13–15]. Since IMRT and VMAT use larger amount of monitor units than a conventional radiotherapy technique such as 3D conformal radiation therapy, in addition to generating a steep dose fall-off between the target volume and nearby OARs, the discordance in dose distributions between the calculation and delivery of IMRT or VMAT could result in critical medical malpractice [16]. Therefore, pre-treatment patient-specific quality assurance (QA) for IMRT and VMAT plans is highly recommended in the clinic as a verification procedure of the treatment plan before patient treatment [16].

As a pre-treatment QA for IMRT and VMAT, 2D gamma evaluation, which is generally performed in the clinic, compares the calculated planar dose distribution in the TPS with the planar dose distribution measured with 2D array dosimeters [17]. For IMRT, 2D gamma evaluation with a gamma criterion of 3%/3 mm has been generally recommended and is routinely applied in the clinic [16]. However, for VMAT, 2D gamma evaluation with a gamma criterion of 2%/2 mm was recommended by Heilemann *et al.* and Fredh *et al.* [18, 19]. They investigated the sensitivity of the 2D global gamma evaluation with 2%/2 mm comparing that with 3%/3 mm to detect errors that were artificially introduced into the VMAT plans. They showed higher sensitivity of the gamma criterion of 2%/2 mm to detect errors in the VMAT plans and recommended its use in the clinic for pre-treatment VMAT QA. For stereotactic ablative radiotherapy (SABR), which should be performed with care owing to its large fraction sizes as well as small fraction numbers, we previously recommended 2D global gamma evaluation with a gamma criterion of 2%/1 mm because of its higher sensitivity in detecting delivery errors in the SABR VMAT plans than those with 2%/2 mm, 1.5%/1.5 mm, and 1%/2 mm [20]. In our previous study, a tolerance level of 90% with a MapCHECK2 (Sun Nuclear Corporation, Melbourne, FL, USA) dosimeter and a tolerance level of 80% with an EBT2 film (Ashland Inc., Covington, KY, USA) was found to be appropriate with the gamma criterion of 2%/1 mm for pre-treatment VMAT QA for SABR.

Although 2D global gamma evaluation with a strict gamma criterion of 2%/1 mm could detect the discordance between the calculated and the measured planar dose distributions better than that with the conventional 2%/2 mm, the setup error of the measurement device could be a detrimental factor in the pre-treatment VMAT QA for SABR [21]. In other words, the gamma-passing rate could be lower than the tolerance level owing to the setup errors of the measurement device, although there is no problem in the SABR VMAT plans. Since the distance to agreement (DTA) of the conventional 2 mm was reduced to 1 mm, 2D gamma evaluation with 2%/1 mm could be more susceptible to setup errors of the measurement device. In the clinic, this could be problematic as it consumes a significant amount of time and human resources. Therefore, we report the results of 2D global gamma evaluation with a gamma criterion of 2%/1 mm for SABR VMAT in this study, which was performed in our institution for a two-year period, from January 2014 to January 2016. Although this report is from a single institution experience, considerable numbers of SABR for a variety of treatment sites with various modulation degrees were performed in our institution; therefore, it could be informative for other clinics to perform VMAT QA for SABR with a gamma criterion of 2%/1 mm.

## RESULTS

### Gamma passing rates and modulation degree of VMAT plans

**Figure 1:**
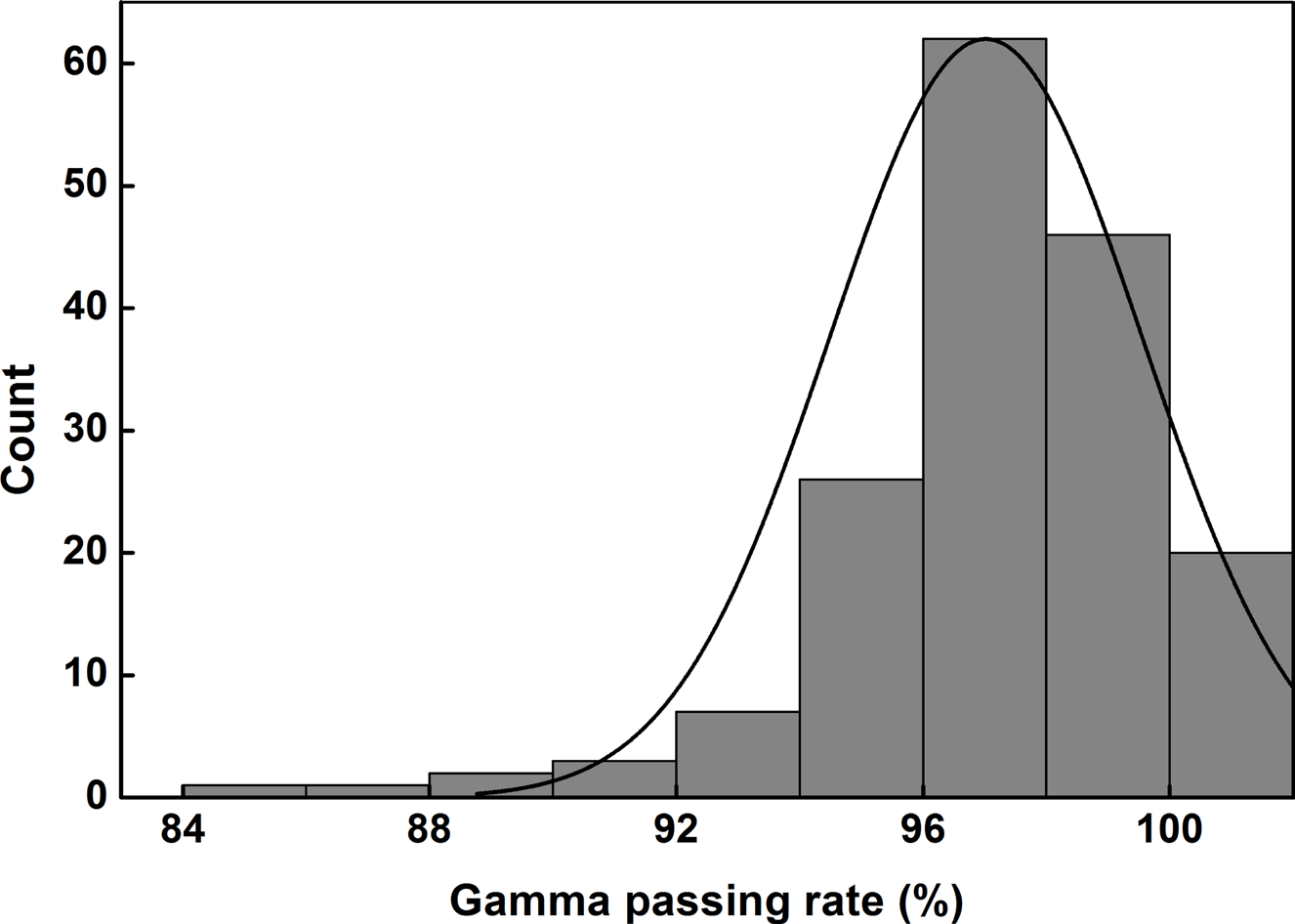
2D global gamma passing rates with a gamma criterion of 2%/1 mm for stereotactic ablative radiotherapy delivered with volumetric modulated arc therapy technique

The distributions of the 2D global gamma-passing rates with 2%/1 mm for SABR VMAT for two years in our institution are shown in Figure 1. The number of cases with a gamma passing rate of 100% with 2%/1 mm was 20 (11.9% of the total examined cases). The number of cases with gamma passing rates greater than or equal to 98%, 96%, 94%, 92%, and 90% was 66 (39.3%), 128 (76.2%), 154 (91.7%), 161 (95.8%), and 164 (97.6%), respectively. From a total of 168 cases, 4 cases (2.4%) failed, with a gamma criterion of 2%/1 mm and a tolerance level of 90%. The gamma passing rates of each treatment site are summarized in Table 1. The average gamma passing rate of lung SABR was the highest among all (97.7% ± 2.4%) while that of C-spine SABR was the lowest among all (94.1% ± 3.2%). No noticeable differences were observed among the gamma passing rates of SABR VMAT plans for each treatment site.

**Table 1:** Details of the analyzed VMAT plan cases for two years

Treatment sites	Total number of cases	Prescription (Gy)	Fraction number (Gy)	MI_s_	%GP with 2%/1 mm (%)
Head & Neck	1	24	3	18.44	99.2
C-spine	6	16 ± 5.4	1 ± 0.0	13.3 ± 3.9	94.1 ± 3.2
T-spine	12	19 ± 3.6	1.3 ± 1.2	10.3 ± 3.6	95.3 ± 3.7
Lung	82	57.0 ± 5.7	4.0 ± 0.3	9.8 ± 2.6	97.7 ± 2.4
Liver	28	39.9 ± 9.6	3.3 ± 0.4	10.5 ± 4.9	97.1 ± 1.8
Abdomen	16	34.4 ± 7.0	3.3 ± 1.0	11.3 ± 8.0	97.1 ± 1.6
Pelvis	9	28.3 ± 5.7	3.3 ± 2.8	11.2 ± 7.5	96.2 ± 1.7
L-spine	12	19.3 ± 3.7	1.2 ± 0.6	12.2 ± 3.5	96.0 ± 2.6
Thorax	2	29.5 ± 13.4	4.0 ± 1.4	22.8 ± 13.4	95.8 ± 3.1
Total	168	43.1 ± 16.4	3.2 ± 1.3	10.7 ± 4.7	97.0 ± 2.5

*Abbreviations*: VMAT, volumetric modulated arc therapy; MI_s_, modulation index evaluating the speed of multi-leaf collimators; %GP with 2%/1 mm, global gamma passing rate with a gamma criterion of 2%/1 mm

**Figure 2:**
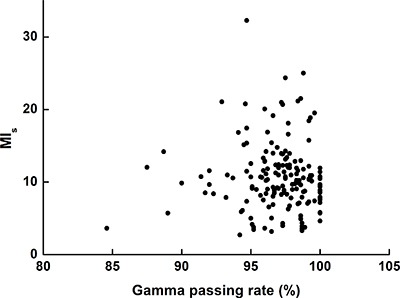
Values of the modulation index evaluating multi-leaf collimator speed (MIs) plotted as a function of the gamma passing rates

The values of the modulation index (MI_s_) in terms of MLC speed are summarized in Table 1 [14]. The MI_s_ values have been plotted as a function of the gamma passing rates with 2%/1 mm in Figure 2. Per the previous study, as the modulation degree increases, the MI_s_ values increase and the gamma passing rates decrease; therefore, the MI_s_ values are inversely proportional to the gamma passing rates [14]. However, this tendency is not observed in Figure 2. Consequently, the Pearson correlation coefficient between the gamma passing rates and the MI_s_ values was 0.014 with a *p* value of 0.861, showing no correlation between the gamma passing rates for SABR VMAT and the MI_s_ values.

### Analysis of the failed cases with a gamma criterion of 2%/1 mm

**Figure 3:**
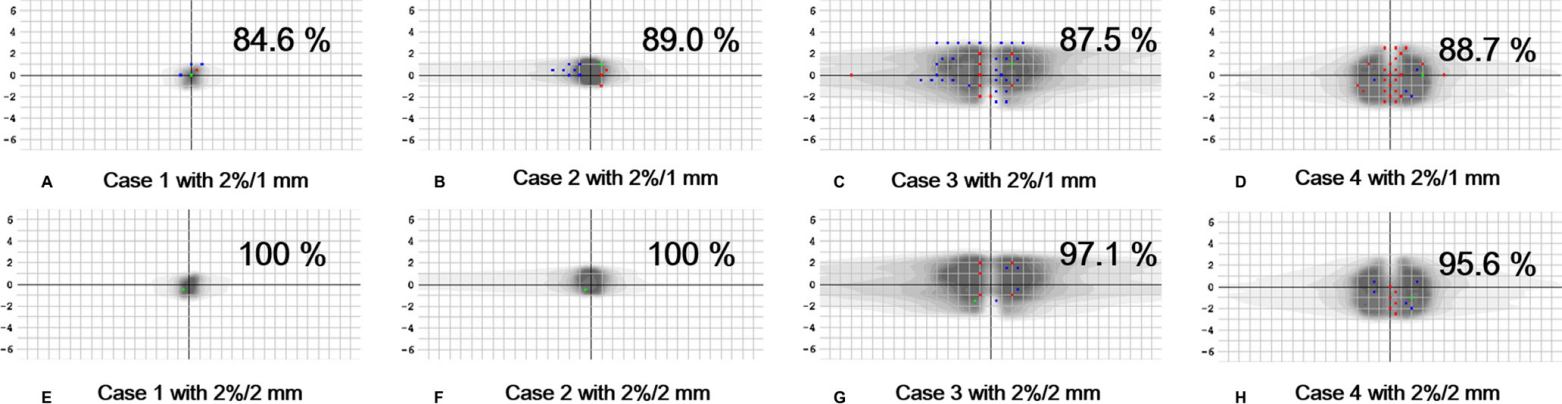
2D global gamma analyses for the four failed stereotactic ablative radiotherapy plans delivered with volumetric modulated arc therapy technioque Points with measured values higher than the calculated values are shown in red dots while points with measured values lower than the calculated values are shin in blue dots.

The details of the four SABR VMAT plans, which failed with a gamma criterion of 2%/1 mm and a tolerance level of 90%, are summarized in Table 2. One lung case and three spine cases failed, showing gamma passing rates less than 90% (89.0% for the lung case, 88.7% for the C-spine case, 87.5% for the L-spine case, and 84.6% for the T-spine case). When the conventional gamma criterion of 2%/2 mm was applied for those four cases, all the gamma passing rates were higher than 95%, as shown in Figure 3.

**Table 2:** Details of the failed cases of the VMAT plans with a gamma criterion of 2%/1 mm and a tolerance level of the global gamma passing rate of 90% for two years

Failed case	Treatment sites	Prescription (Gy)	Fraction number	MI_s_	%GP with 2%/1 mm	%GP with 2%/2 mm
1	T-spine	13	1	3.63	84.6	100
2	Lung	60	4	5.73	89.0	100
3	L-spine	20	1	12.03	87.5	97.1
4	C-spine	16	1	14.18	88.7	95.6

*Abbreviations*: VMAT, volumetric modulated arc therapy; MI_s_, modulation index evaluating the speed of multi-leaf collimator; %GP with 2%/1 mm, global gamma passing rate with a gamma criterion of 2%/1 mm; %GP with 2%/2 mm, global gamma passing rate with a gamma criterion of 2%/2 mm.

The MI_s_ values of the four failed cases ranged from 3.63 to 14.18. The MI_s_ values of the lung and T-spine cases were 3.63 and 5.73, respectively, which were much lower than the average MI_s_ value (10.7) calculated from the wholly examined cases (168 cases). The MI_s_ values of the L-spine and C-spine cases were 12.03 and 14.18, respectively, which were slightly higher than the average MI_s_ value. However, the MI_s_ values of the L-spine and C-spine cases constituted the top 26.8% and the top 15.8% of the all the examined cases, respectively.

The changes in the gamma passing rates of the failed cases after simulating setup errors, which included yaw rotational setup errors and translational setup errors, in the left-to-right (LR), superior-to-inferior (SI), and anterior-to-posterior (AP) directions are summarized in Table 3. For the T-spine SABR VMAT plan, no improvement in the gamma-passing rate was observed by simulating setup errors. However, for the rest of the failed cases, gamma-passing rates were greater than 90% with a shift of 1 mm. For the lung and L-spine SABR VMAT plan, shifting the measurement device by 1 mm in the LR direction increased the gamma passing rates to 100% and 91.6%, respectively. For the C-spine SABR VMAT plan, by shifting the measurement device by 1 mm in the AP direction, the passing rate increased to 94.2%.

**Table 3:** Changes in gamma passing rates of the failed cases by simulating setup errors

		Gamma passing rate with 2%/1 mm (%)		
Couch rotation	–0.2°	–0.1°	0.1°	0.2°
T-spine	84.6	84.6	84.6	84.6
Lung	89.4	89.5	87.2	87.2
L-spine	87.3	87.9	87.8	87.5
C-spine	88.6	88.6	88.4	88.9
SI shift	–0.2 cm	–0.1 cm	0.1 cm	0.2 cm
T-spine	83.0	84.6	84.6	84.6
Lung	82.2	89.0	89.0	85.5
L-spine	88.4	87.5	87.5	86.0
C-spine	88.0	88.7	88.7	86.2
LR shift	–0.2 cm	–0.1 cm	0.1 cm	0.2 cm
T-spine	82.7	84.6	84.6	84.1
Lung	*97.5*	*100*	87.6	85.5
L-spine	*90.4*	*91.6*	87.5	85.5
C-spine	85.5	88.7	88.7	86.0
AP shift	–0.2 cm	–0.1 cm	0.1 cm	0.2 cm
T-spine	73.1	79.2	57.6	24.3
Lung	64.0	73.3	55.6	54.5
L-spine	82.6	86.2	85.6	81.4
C-spine	81.7	84.7	*94.2*	*93.2*

*Abbreviations*: SI, superior to inferior direction; LR, left to right direction; AP, anterior to posterior direction

Note: The gamma passing rates higher than 90% are shown in italic.

The changes in the gamma passing rates of the failed cases and the number of tested points during gamma evaluation in terms of the threshold values are shown in Table 4. The number of tested points increased as the threshold values decreased. The T-spine, lung, and C-spine SABR VMAT plans showed higher gamma passing rates than the tolerance level of 90% with a threshold value of 5%. In the case of the T-spine plan, the gamma-passing rate did not change with the setup error simulations; however, it changed to 92.2% upon lowering the threshold value to 5%. In this case, the number of tested points increased from 26 to 51 by changing the threshold value from 10% to 5%, which was a drastic increase (96.2%) in the number of the tested points.

Table 4Changes in gamma passing rates and number of tested points of the failed cases with increasing threshold values

**Table:** 

	Target volume size (cc)	Gamma passing rate with 2%/1 mm (%)					
		5%	10%	15%	20%	25%	30%
T-spine	0.5	*92.2*	84.6	77.8	73.3	80.0	80.0
Lung	3.2	*92.0*	89.0	85.1	76.2	71.4	71.0
L-spine	153.7	89.2	87.5	87.1	87.5	87.0	85.4
C-spine	75.7	*91.0*	88.7	84.2	79.9	76.4	73.6

**Table:** 

		No. of tested points during gamma evaluation					
		5%	10%	15%	20%	25%	30%
T-spine	0.5	51	26	18	15	10	10
Lung	3.2	125	91	67	42	35	31
L-spine	153.7	351	305	278	256	246	206
C-spine	75.7	310	247	184	139	123	106

Note: The gamma passing rates higher than 90% are shown in italic.

## DISCUSSION

In this study, we demonstrated the gamma evaluation results for SABR VMAT with a gamma criterion of 2%/1 mm from January 2014 to January 2016. For two years, a total of 168 cases of gamma evaluations for SABR VMAT plans were carried out and four of them failed with 2%/1 mm and 90% tolerance level (failure rate of 2.4%) [20]. The decision to use the gamma criterion of 2%/1 mm rather than 2%/2 mm was meant to reduce the DTA from 2 mm to 1 mm. This could increase the sensitivity of the gamma evaluation in detecting errors in the treatment plans; however, it makes the gamma evaluation more susceptible to setup errors of the measurement device, which is impractical in the clinic. The results of this study revealed that no concerns are necessary in the use of 2%/1 mm with a 90% tolerance level since only four of the 168 cases failed with gamma evaluation using the 2%/1 mm gamma criterion for SABR VMAT. The gamma evaluation with 2%/1 mm was successfully applied in the clinic for the pre-treatment patient-specific VMAT QA for SABR in our institution for two years.

The calculation resolution of the reference 2D dose distribution was 1 mm while the detector resolution of the MapCHECK2 dosimeter was 7.07 mm in the diagonal direction. Some previous studies showed that the grid size of the detector array could affect the gamma passing rates, therefore, the results in this study is only valid for the MapCHECK2 dosimeter or detector arrays with similar detector resolutions to that of the MapCHECK2 dosimeter [22, 23]. Since those previous studies recommended the calculation grid size should be smaller than the mearement grid size, we used calculation grid size of 1 mm in this study [22, 23].

Previously, Park *et al.* showed considerable correlations with statistical significance between the MI_s_ values and gamma passing rates [14]. In that study, as the modulation degree of the VMAT plans increased, the gamma passing rates decreased and the values of MI_s_ increased. Upon reviewing the four failed cases of our study, we found that the MI_s_ values of these cases were not particularly high; therefore, the failures with the gamma evaluation were apparently not caused by the high modulation of the VMAT plans. The gamma passing rates of the other SABR VMAT plans, which were acquired in the same week in which the failed cases were acquired, showed higher gamma passing rates than the tolerance level. Therefore, no systemic errors existed in the linac delivery system or in the TPS. When we applied gamma evaluation with 2%/2 mm, *i.e.*, increased the DTA, the passing rates of those failed cases became greater than 95%. In this respect, we assumed that the setup errors of the measurement device may have caused the low passing rates [21]. By simulating setup errors, *i.e.*, shifting the measuring device by 1 mm in the LR direction, the VMAT plans for the lung SABR and the L-spine SABR showed gamma passing rates greater than 90% by. In the case of the VMAT plan for C-spine SABR, by shifting the measuring device by 1 mm in the AP direction, a passing rate of 94.2% was achieved. However, there was no improvement in the gamma-passing rate of the VMAT plan for T-spine SABR with the setup error simulation. We observed that the number of the tested points during gamma evaluation for the T-spine SABR VMAT was extremely small, *i.e.*, only 26 points. Therefore, we decreased the threshold value to 5%, and the number of the tested points increased to 51 (a 96% increase). Subsequently, we achieved 92.2% passing rate with the 2%/1 mm for the T-spine SABR VMAT. Besides the T-spine VMAT, the VMAT plans for lung SABR and C-spine SABR showed gamma passing rates greater than 90% when the threshold value was changed to 5%. The number of tested points of the VMAT plans for the lung SABR and C-spine SABR increased by 37% and 26%, respectively, when the threshold value was changed to 5%. We observed a decreasing tendency in the gamma passing rates with increasing threshold values, which was in agreement with the results in the study by Steers *et al.*, which showed increased sensitivity of the gamma evaluation with increasing threshold values [24]. Therefore, the low gamma-passing rate of the VMAT plan for T-spine SABR may have been caused by the small number of tested points, since the gamma-passing rate is a percent value. After searching the error source for each of the four failed cases, we verified the four VMAT plans with regard to their deliverability for patient treatment.

Several studies recently showed irrelevance of the 2D gamma passing rates toward the changes in the clinically relevant dose-volumetric parameters between the treatment plan and actual delivery [22, 25]. Information of the 2D gamma evaluation is not enough to detect errors affecting delivered doses which are three-dimensional inside a patient's body [26]. Moreover, since gamma evaluation is a comprehensive evaluation tool for a plan as a whole and not an evaluation tool for the delivered doses to each organ individually, the dose delivery accuracy to each structure including the target volumes and OARs, could not be verified individually with gamma evaluation [13]. Despite these limitations, 2D gamma evaluation is still performed in the clinic widely because no better alternative exists. To overcome the limited information of the 2D gamma evaluation, 3D gel dosimeters were proposed for the pre-treatment patient-specific QA for IMRT or VMAT [27]. However, the accuracy of the various 3D gel dosimeters is not adequate to be used in the clinic yet, owing to the high uncertainty. Some dosimeters recently introduced in the clinic are capable of measuring quasi-3D dose distributions, such as COMPASS™ system (IBA Dosimetry GmbH, Schwarzenbruck, Germany), ArcCHECK™ (Sun Nuclear Corporation, Melbourne, FL, USA), and OCTAVIUS 4D™ system (PTW, Freiburg, Germany) [22, 26, 28]. Although these dosimetry systems can provide more information than the 2D dosimeters, they have their own limitations. Although the COMPASS system can reconstruct dose distributions with actually measured fluences during beam delivery, the dose distribution is reconstructed with its own dose calculation algorithm. Therefore, this is not fully based on the measurement [26]. For the ArcCHECK system, the measured fluence could be used for the reconstruction of the dose distribution in the patient CT image with the 3DVH™ software (Sun Nuclear Corporation, Melbourne, FL, USA). However, this system also has the same limitation as that of the COMPASS system [28]. In the case of OCTAVIUS 4D system, the delivered-dose discrepancy in individual organs cannot be evaluated since that system does not utilize patient CT images [22]. Moreover, these quasi-3D dosimetry systems are not available to all radiotherapy institutions. On the other hand, some suggested the utilization of linac log files for the pre-treatment QA, which are recorded in the linac operating system during the actual delivery of a plan [29]. This method has an intrinsic disadvantage as a verification method for IMRT or VMAT plans because it is not an independent verification method, *i.e.*, the accuracy of the delivered doses accomplished with the radiotherapy system including the linac and the TPS is verified with the same system. In this respect, 2D gamma evaluation is still used generally in the clinic. Therefore, enhancing the performance of 2D gamma evaluation has some merits [18–20, 24].

We previously suggested a gamma criterion of 2%/1 mm for pre-treatment SABR VMAT QA rather than 2%/2 mm to enhance the sensitivity of gamma evaluation in detecting errors in the SABR VMAT plans [20]. There might be some concern regarding the use of the gamma criterion of 2%/1 mm in the clinic because it could make the gamma evaluation susceptible to setup errors during measurements. However, as shown in the results of this study, the gamma evaluation with 2%/1 mm could be successfully applied in the clinic. Based on our two-year experience, use of the gamma criterion of 2%/1 mm for SABR VMAT can be easily adopted in the clinic while enhancing the sensitivity of the gamma evaluation to detect errors in a treatment plan.

## MATERIALS AND METHODS

### VMAT plan information

168 VMAT plans for SABR were generated and delivered to patients from January 2014 to January 2016 in our institution. The treatment plan details are summarized in Table 1. The target volumes of SABR were located in the lungs (82 cases), spine (30 cases), liver (28 cases), abdomen (16 cases), pelvis (9 cases), thorax (2 cases), and the head and neck (1 case). The prescription doses ranged from 6 Gy to 60 Gy and the fraction sizes ranged from 3 Gy to 24 Gy. The fraction number ranged from 1 to 10. Every patient underwent CT scans with a Brillance CT Big Bore™ (Philips, Amesterdam, Netherlands). During CT scans, patients were immobilized with appropriate immobilization devices compatible for each treatment site. For the treatment sites such as the lung, where respiratory motion is considerable, the motion was minimized with a Body Pro-Lok system (CIVICO, Orange City, IA, USA) and internal target volumes (ITVs) were defined with the 4D CT. For SABR, the ITV, rather than the respiratory gating technique, was used. All the VMAT plans were generated with an Eclipse™ system using the TrueBeam STx™ with a high-definition MLC (Varian Medical Systems, Palo Alto, CA, USA). According to the treatment site, a 6 MV flattening filter free (FFF) photon beam or 10 MV FFF photon beam was used. For optimization of the VMAT plans, a progressive resolution optimizer (PRO3, version 10, Varian Medical Systems, Palo Alto, CA, USA) was used. For the calculation of dose distributions, the anisotropic analytic algorithm (AAA, version 10, Varian Medical Systems, Palo Alto, CA, USA) was used. Except for lung SABR, a dose calculation grid of 2 mm was used for all the other cases. In the case of lung SABR, a dose calculation grid of 1 mm was used [30].

### 2D global gamma evaluation with a gamma criterion of 2%/1 mm

Before treatment, every SABR plan was verified using the 2D global gamma-index method with absolute doses by measuring planar dose distributions with the MapCHECK2 dosimeter. The MapCHECK2 dosimeter is a detector array with a total of 1527 solid state diodes. The diagonal detector spacing was 7.07 mm and the detector spacing parallel to X and Y axes were both 10 mm. Unlike the MapCHECK dosimeter, the MapCHECK2 dosimeter is compatible with VMAT using the Isocentric Mounting Fixture™ or MapPHAN™ (Sun Nuclear Corporation, Melbourne, FL, USA). In this study, the MapCHECK2 dosimeter was inserted into the MapPHAN which is a solid water phantom with a hole for insertion of the MapCHECK2. For the calculation of reference dose distributions, CT images of the MapCHECK2 inserted into the MapPHAN were acquired with a slice thickness of 1 mm. With these CT images, the reference planar dose distributions were calculated with the Eclipse system with a calculation grid of 1 mm. The MapCHECK2 dosimeter was calibrated every month. When measuring 2D dose distributions, the MapCHECK2 inserted into the MapPHAN was setup with a light field and room laser system in the treatment room. After measuring dose distributions for each SABR VMAT plan, 2D gamma evaluations were performed with the SNC patient software (version 6.1.2, Sun Nuclear Corporation, Melbourne, FL, USA). The global gamma evaluation with a gamma criterion of 2%/1 mm was performed with absolute doses. The points with doses less than 10% of the maximum measured dose were ignored for the gamma evaluation, *i.e.,* the threshold value was set as 10%.

### Evaluation of modulation degree

To examine the correlation of the gamma passing rate to the modulation degree, we calculated the MI_s_ [14]. The MI_s_ values were calculated with the DICOM-RT formatted treatment plan files exported from the Eclipse system. For correlation analysis, the Pearson correlation coefficient was calculated between gamma passing rates and the MI_s_ values.

### Analysis of the failed cases

Among 168 tested cases with the 2D global gamma evaluation, the failed cases with a tolerance level of 90% were analyzed. We traced the reason of the failure with gamma evaluation with 2%/1 mm for the failed cases by simulating setup errors and changing the threshold values. We simulated setup errors by rotating the couch from −0.2° to 0.2° (yaw rotational error) at intervals of 0.1°, translating the couch from −2 mm to 2 mm at intervals of 1 mm in the LR, SI, and AP directions. We examined the changes in the gamma passing rates by changing the threshold values from 5% to 50% at intervals of 5%.

## Abbreviations

IMRT: intensity modulated radiation therapy
VMAT: volumetric modulated arc therapy
OAR: organ at risk
MLC: multi-leaf collimator
TPS: treatment planning system
MU: monitor unit
3D CRT: 3D conformal radiation therapy
QA: quality assurance
SABR: stereotactic ablative radiotherapy
DTA: distance to agreement
MI: modulation index
LR: left-to-right
SI: superior-to-inferior
AP: anterior-to-posterior
ITV: internal target volume
FFF: flattening filter free
PRO: progressive resolution optimizer
AAA: anisotropic analytic algorithm
